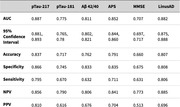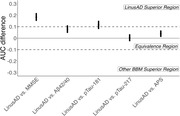# Early Identification of Alzheimer’s Disease with a Machine Learning‐Enabled Digital Cognitive Assessment: Concurrent Detection of Cognitive Impairment and Amyloid‐Beta PET Status

**DOI:** 10.1002/alz.093435

**Published:** 2025-01-09

**Authors:** Ali Jannati, Karl Thompson, Claudio Toro‐Serey, Connor Higgins, Russell Banks, John Showalter, David Bates, Sean Tobyne, Alvaro Pascual‐Leone

**Affiliations:** ^1^ Linus Health, Boston, MA USA; ^2^ Harvard Medical School, Boston, MA USA; ^3^ Michigan State University, East Landing, MI USA; ^4^ Hebrew SeniorLife, Boston, MA USA

## Abstract

**Background:**

Maximizing the benefits of disease‐modifying treatments (DMTs) for Alzheimer’s disease (AD) requires early identification of cognitive impairment and abnormal brain amyloid‐beta (Aβ) status. Either one alone is insufficient. Additionally, clinical trials of DMTs are impeded by high screen failure rates and costly prescreening. Thus, an efficient and cost‐effective solution to streamline the process of early AD identification is urgently needed. This study aimed to assess the accuracy of a brief digital cognitive assessment, the Linus Health Digital Clock and Recall (DCR), to identify cognitive impairment and predict brain Aβ status.

**Method:**

930 participants (mean age 72.0±6.7; 56.8% female; 23% minorities) in the Bio‐Hermes‐001 study were classified as cognitively unimpaired, mild cognitive impairment, or probable Alzheimer’s dementia, and 35.1% were Aβ+ on 18F‐florbetapir PET scan. A DCR‐based algorithm (LinusAD) used age, APOE status, drawing metrics, speech and acoustic features, and temporal‐spatial features of stylus manipulation. LinusAD was compared with MMSE and blood‐based biomarkers (BBMs) Lilly pTau‐217, Quanterix pTau‐181, C2N Aβ42/40, and C2N Amyloid Probability Score (APS). Superiority or non‐inferiority was established if 95% confidence interval of the bootstrapped AUC difference between two tests was higher than or within ΔAUC±0.1.

**Result:**

Cognitive‐impairment identification by the DCR (AUC=0.85) was superior to Aβ42/40, pTau‐181, and pTau‐217 (AUCs=0.63, 0.66, 0.72) and non‐inferior to RAVLT and MMSE (AUCs=0.89, 0.82). Aβ‐status prediction by LinusAD (AUC=0.882) was equivalent to BBMs (AUC range: 0.775–0.887; average AUC=0.82) and superior to MMSE (AUC=0.71) (see Table and Figure). Combining the DCR with Aβ42/40, pTau‐181, and pTau‐217 improved their Aβ‐status prediction performance to AUCs of 0.882, 0.835, and 0.905, respectively.

**Conclusion:**

The 3‐minute DCR was superior to BBMs in detecting cognitive impairment and boosted their Aβ‐PET prediction ability to levels comparable to CSF biomarkers of AD. Digital cognitive assessments that leverage AI process metrics, such as the DCR, enable cost‐effective integration into multi‐step clinical workflows to prioritize the most suitable patients for BBM testing, DMTs, and decrease high screen‐failure rates in AD clinical trials.